# Molecular regulators of exercise‐mediated insulin sensitivity in non‐obese individuals

**DOI:** 10.1111/jcmm.18015

**Published:** 2023-11-08

**Authors:** Shamma Almuraikhy, Asmaa Doudin, Alexander Domling, Asmaa Ali J. F. Althani, Mohamed A. Elrayess

**Affiliations:** ^1^ Biomedical Research Center Qatar University Doha Qatar; ^2^ Groningen Research Institute of Pharmacy, Drug Design Groningen University Groningen The Netherlands; ^3^ Department of Biomedical Sciences, College of Health Science, QU Health Qatar University Doha Qatar; ^4^ College of Pharmacy, QU Health Qatar University Doha Qatar

**Keywords:** creatine kinase (CK), glutathione (GSH), hepatic insulin‐sensitizing substance (HISS), high‐intensity interval (HIT), malondialdehyde (MDA), peroxisome proliferator‐activated receptor alpha‐stimulated lipid mediators (PAHSAs), short‐chain fatty acids (SCFAs), superoxide dismutase (SOD), thiobarbituric acid reactive substances (TBARS), total antioxidant status (TAS), type 2 diabetes mellitus (T2DM)

## Abstract

Insulin resistance is a significant contributor to the development of type 2 diabetes (T2D) and is associated with obesity, physical inactivity, and low maximal oxygen uptake. While intense and prolonged exercise may have negative effects, physical activity can have a positive influence on cellular metabolism and the immune system. Moderate exercise has been shown to reduce oxidative stress and improve antioxidant status, whereas intense exercise can increase oxidative stress in the short term. The impact of exercise on pro‐inflammatory cytokine production is complex and varies depending on intensity and duration. Exercise can also counteract the harmful effects of ageing and inflamm‐ageing. This review aims to examine the molecular pathways altered by exercise in non‐obese individuals at higher risk of developing T2D, including glucose utilization, lipid metabolism, mitochondrial function, inflammation and oxidative stress, with the potential to improve insulin sensitivity. The focus is on understanding the potential benefits of exercise for improving insulin sensitivity and providing insights for future targeted interventions before onset of disease.

## 1 INTRODUCTION

According to the International Diabetes Association, there is a global rise in diabetes incidence accounting for 425 million cases especially in individuals aged 20–79 years, while it is estimated that the number of people with diabetes will reach 463 million in 2030 according to the World Health Organization (WHO).^1^ The most common risk factors of T2D are being overweight or obese, high blood pressure and glucose levels, age, dietary factors such as high‐fat and high‐sugar diets, sedentary lifestyle and urbanization.^1^ Particularly, obesity plays a major role in increasing the risk of T2D and insulin resistance (IR).^2^, ^3^, ^4^ Further to the established link between obesity and IR, multiple studies have indicated that apparently healthy non‐obese subjects can also develop T2D and IR.^5^ The development of insulin resistance in non‐obese subjects is similarly associated with a variety of factors, including genetic predisposition, lifestyle factors, and metabolic abnormalities including glucose metabolism, lipid metabolism and inflammatory pathways. Therefore, prevention of T2D in both non‐obese and obese at‐risk individuals entails structured intensive lifestyle changes, including physical exercise and diet.^3^, ^6^ Numerous studies have consistently demonstrated the significant benefits of regular physical exercise on blood sugar regulation and cardiovascular health in the general population. In fact, research has indicated that individuals who engage in regular physical activity have a significantly lower risk of developing cardiovascular diseases.^7^ Additionally, long‐term physical activity has been shown to not only improve blood sugar control in diabetic patients but also significantly enhance their overall quality of life.^7^, ^8^, ^9^ Other positive health outcomes include improved insulin sensitivity and reduced risk of T2D. These benefits are thought to be partially due to the anti‐inflammatory and antioxidative stress effects of exercise, which are regulated by various molecular mediators (Table 1). Despite the potential importance of these mediators in the protective effects of exercise, their role in insulin resistance and the prevention of T2D is not fully understood. In this review, we aim to better understand the molecular mediators of insulin resistance in non‐obese individuals and explore potential strategies for preventing insulin resistance and T2D.^23^

**TABLE 1:** Molecular mediators that play a role in the insulin‐sensitizing effect of exercise in muscle tissue.

Model	Findings	Ref
*Modulation of oxidative stress*		
15 Healthy adults completed a cycling exercise until exhaustion	Exercise until exhaustion resulted in an increase in oxidative stress, as indicated by rising levels of TBARS and reduced levels of glutathione	[10]
18 sedentary participants exercised for 40 minutes at moderate and severe intensities	Heavy exercise reduces lymphocyte GSH levels, which increases oxidative stress‐induced apoptosis. However, moderate exercise reduces oxidative stress‐induced apoptosis by improving the cell's antioxidant capacity	[11]
51 sedentary men were divided into four groups (1 control and 3 exercise) to study the effect of different intensities of aerobic exercise on serum levels of MDA and CK	Single‐session aerobic exercise increase the levels of MDA and CK in sedentary males. Low‐intensity exercise may be more beneficial to prevent lipid peroxidation and muscle damage	[12]
18 male soccer players were divided into three groups based on the intensity level of their 60‐minute training: low, medium, and high intensity	The oxidant/antioxidant balance in lymphocytes and neutrophils is influenced by exercise intensity, with high intensity leading to oxidative damage in lymphocytes	[13]
Healthy adults completed a cycling exercise until exhaustion	TBARS, GSH are superior markers of oxidative stress alterations during both dynamic and static physical activity, while TAS is unreliable in detecting exercise‐generated oxidative stress during isometric exercises	[14]
Rats subjected to experimental heart injury, then divided into three groups and observed for 3 months: sedentary control, low‐intensity training and high‐intensity interval training (HIT) groups	HIT and low‐intensity training improves heart function and reduces oxidative stress compared to sedentary behaviour. Continuous low‐intensity training shows impairment of heart function	[15]
After a 4‐week intervention, involving nine male triathletes and six sedentary men, a duathlon was conducted by the triathletes. Blood samples were collected from all participants both before and after the intervention	After 4 weeks of intensive exercise, levels of TBARS increased and total antioxidant levels decreased in both active and sedentary individuals	[16]
*Modulation of inflammatory responses*		
29 subjects aged 18–35 and 31 aged 65–85 were divided into 4 groups: young physically active (YPA), young physically Inactive (YPI), old physically active (OPA) and old physically inactive (OPI). The inactive groups underwent a 12‐week programme of aerobic and resistance exercises, while the active control groups continued their normal exercise regimen. Blood samples were taken before and after the 12‐week period	Serum CRP levels decreased in the YPI and OPI groups with training. Plasma IL‐6 and IL‐1beta remained unchanged, but TNF‐alpha was higher at baseline and after the intervention period for YPI and YPA	[17]
88 elite male athletes from diverse sports disciplines	The levels of IL‐10 and markers of inflammation and oxidative stress vary in different sports disciplines and can be used as potential indicators of an athlete's health, performance, and recovery from injury. In moderate‐power sports, IL‐10 levels were higher compared with high‐power sports, but SOD and MDA levels were higher in high‐power sports. In endurance sports, IL‐10 and MIP‐1beta increased in low/moderate endurance compared with high endurance	[18]
*Modulation of metabolic pathways*		
55 sedentary older women were included in the physical activity programme	Exercise stimulated the production of PAHSAs, which have insulin‐sensitizing effects	[19]
A T2DM mice with high‐fat diet and injections of streptozocin	Exercise increased the levels of SCFAs in the plasma	[20]
9 men underwent euglycaemic hyperinsulinaemic clamp during recovery from a one‐legged knee‐extensor workout. Muscle biopsies were taken to obtain pools of type I and type II fibres	A single bout of exercise increased the insulin sensitivity of both type I and type II muscle fibres	[21]
Exercise on a running wheel in ageing rats exercise on a running wheel	In ageing rats, exercise has been found to improve insulin sensitivity through the restoration of HISS action	[22]

## 2 INSULIN RESISTANCE IN APPARENTLY HEALTHY NON‐OBESE INDIVIDUALS

**FIGURE 1:**
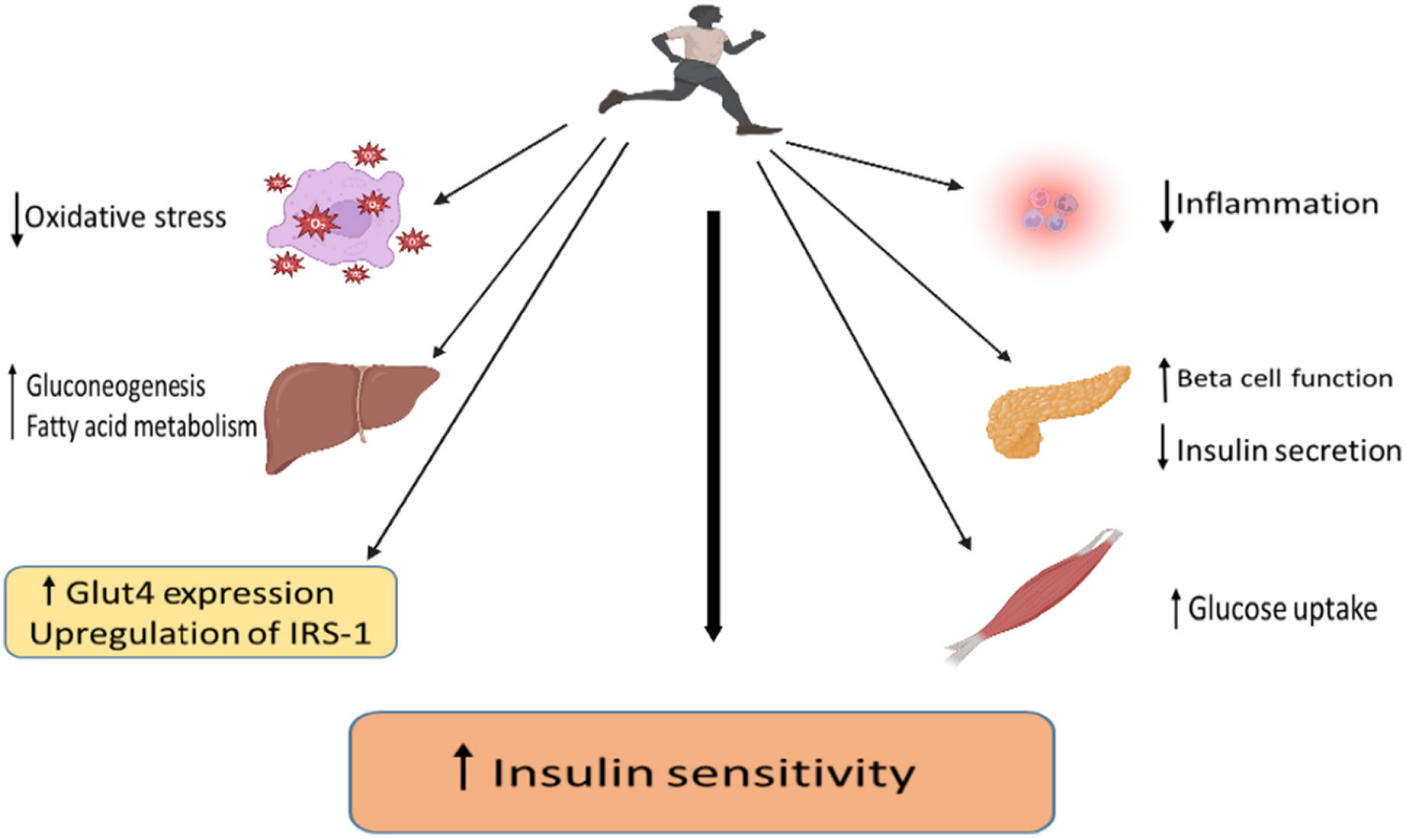
Exercise‐mediated insulin sensitivity and its effect on inflammation and oxidative stress.

Individuals with insulin resistance are characterized by the inability to respond to secreted insulin, which can be at normal or high levels.^24^ IR is characterized by an abnormal interaction between skeletal muscle, liver and adipose tissue, which can lead to an increase in free fatty acids and impaired glucose uptake and production (gluconeogenesis) (Figure 1). This can result in high blood sugar levels and weight gain, particularly around the waist. It. Evidence suggests that an increase in body weight by more than 35% can lead to a reduction in insulin sensitivity in multiple organs, promoting IR and increasing the risk of T2D.^25^, ^26^, ^27^ Individuals with a body mass index (BMI) between 20 and 27 kg/m^2^, who are either lean or overweight, and who have gained 2 to 10 kg of body fat mass during adulthood, are at an increased risk of developing insulin resistance and T2D The prevalence of insulin resistance among apparently healthy non‐obese individuals is estimated to be between 10 and 20 per cent. The term ‘metabolically obese, but normal‐weight’ (MONW) includes non‐obese individuals with normal body weight, b with high‐fat and triglyceride levels.^28^, ^29^ The MONW individuals have an increased risk of hyperinsulinemia,^30^ myocardial infraction^31^, ^32^ and metabolic disorders.^33^, ^34^ According to a recent study, over 40% of lean and overweight females under the age of 30 in Qatar are IR,^35^ compared to 25% of overweight females in other ethnicities.^36^, ^37^, ^38^ It has been suggested in previous reports that insulin resistance can be used as a marker to predict up to 80% of cases of T2D in non‐obese individuals.^38^ It has also been indicated that over 30% of overweight individuals in Qatar are at risk of developing T2D.^39^ A recent study of young, lean individuals with an average BMI less than 22.5 found that both normotensive and hypertensive non‐obese individuals had higher levels of plasma insulin and were IR (*n* = 148).^40^, ^41^, ^42^ Other studies have shown that non‐obese, normoglycemic individuals and/or first‐degree relatives of T2D patients had hyperinsulinemia and IR, suggesting a genetic component.^43^, ^44^ Generally, insulin resistance in non‐obese subjects can be identified using glucose tolerance tests and measurements of insulin, lipids, blood pressure, BMI and waist circumference.^45^ However, despite of the high prevalence of MONW group and its association with a high risk of T2D, it can be very challenging to identify and diagnose those at a higher risk in a timely and accurate manner. Identifying individuals in the MONW group allows for early intervention measures, including diet and exercise, which are essential for prevention and early treatment of T2D.^33^, ^45^, ^46^

## 3 EXERCISE AS A NON‐PHARMACEUTICAL INTERVENTION FOR IMPROVING INSULIN RESISTANCE AND T2D OUTCOMES

Several investigations indicate that diet and physical activity can help reduce the risk of diabetes^47^, ^48^ and offer a feasible practical solution to primary healthcare systems to use in managing glycaemic control among T2D patients.^48^, ^49^ Accumulating evidence suggests that physical training improves glycaemic control and blood lipids profile and decreases the risk of IR as well as T2D health‐associated outcomes.^50^, ^51^ Engaging in regular physical activity can also improve muscular strength, aerobic capacity, endothelial function and body composition.^26^ Regular exercise training, especially aerobic training, has been found to improve cardiovascular and pulmonary function as well as circulating glucose levels.^52^ Given the significant body of evidence supporting the use of exercise in the treatment and management of T2D, the American College of Sports Medicine has developed structured exercise programmes specifically for individuals at high risk of insulin resistance.^53^ Particularly, T2D patients are recommended to exercise for at least 150 minutes per week at a target heart rate of 70% of maximal heart rate and engage in activities such as walking and jogging on a regular basis. Walking in particular has been shown to improve glucose metabolism and cardiorespiratory fitness, leading to improved outcomes and quality of life in IR individuals.^54^ In addition to its other benefits, reducing sedentary behaviour and increasing physical activity can lower body fat percentage, leading to improvements in blood pressure and lipid profile, which can in turn reduce the risk of cardiovascular disease.^55^

In non‐obese individuals with impaired fasting glucose and glucose tolerance, 5 years of diet and training were reported to reduce the risk of T2D, with more profound effects in younger non‐obese subjects compared with older obese counterparts.^56^ Additionally, school‐based physical activity in non‐obese students was shown to play an important role in improving both body composition and insulin sensitivity and in decreasing inflammation.^57^ Other recent studies in normal‐weight rats^58^, ^59^ and humans^60^ showed that skeletal muscle sensitivity to insulin has increased after acute exercise and physical training. Regular exercise in IR non‐obese individuals was shown to improve glucose tolerance and insulin sensitivity, as well as various lipid parameters, blood pressure and fibrinolytic activity.^60^, ^61^, ^62^ Interestingly, even exercise programmes that produced no or little change in VO_2_ max, such as long‐term walking, reported improvement in insulin sensitivity.^63^, ^64^ Moreover, Meyer‐Davis et al showed a negative correlation between weight changes and baseline fasting insulin levels over 5 years.^65^ Another study in lean and overweight T2D patients showed that exercise with diet therapy over a 6‐year period can decrease glucose tolerance impairment by 50% and the progression to overt T2D by 30–50%.^66^ Another study in non‐obese T2D Japanese patients showed that after 7 days of low‐intensity training and diet control, IR as well as triglycerides and fasting blood sugar levels were significantly reduced.^67^ A similar study on the effect of aerobic training in non‐obese T2D individuals showed that there was significant reduction in waist and hip circumference and BMI compared with control group, in addition to a decrease in IR, blood glucose and insulin levels.^68^

## 4 PHYSICAL ACTIVITY CAN INDUCE LONG‐TERM ADAPTATIONS IN CELLULAR METABOLISM AND IMMUNE CELLS, INCLUDING DECREASED OXIDATIVE STRESS AND PRO‐INFLAMMATORY CYTOKINES LEVELS

Overall, physical activity can induce long‐term adaptations in cellular metabolism and immune cells, including decreased oxidative stress status and pro‐inflammatory cytokines levels with a concomitant rise in antioxidants and anti‐inflammatory factors.^69^ Moderate physical and training can be considered as an effective strategy for changing the balance between pro‐ and antioxidant products.^70^ Investigating the relationship between oxidative stress, inflammation and hyperglycaemia is essential to understand the underlying molecular mechanisms and to treat prediabetes at an early stage.^5^ Figure 1 elucidates the mechanisms underlying the augmentation of insulin sensitivity through exercise, along with its consequential effects on inflammation and oxidative stress.

### 4.1 Moderate exercise decreases oxidative stress and improves antioxidant status, while intense exercise increases oxidative stress in the short term

Moderate exercise is defined as a physical activity that requires moderate levels of effort and is generally performed for 30–60 minutes per day. There is a growing body of evidence suggesting that moderate exercise can have a number of beneficial effects on the body, including improving cardiovascular health, reducing the risk of chronic diseases and promoting mental well‐being. One potential mechanism by which moderate exercise may exert these benefits is through its effects on oxidative stress. Many studies have highlighted that moderate exercise decreases oxidative stress and increases antioxidant status. For example, glutathione reductase levels increased after moderate exercise (70% VO2max) in young sedentary obese subjects; however, no change is found in non‐obese and moderate obese people.^70^ In summary, the literature suggests that moderate exercise may be associated with a decrease in oxidative stress and an improvement in antioxidant status in healthy individuals. However, more research is needed to fully understand the relationship between moderate exercise and oxidative stress and to determine how it may vary in different populations and health status. The relationship between intense exercise and oxidative stress is complex and may depend on a number of factors, including the duration and intensity of the exercise, the type of exercise and individual characteristics such as age, sex and fitness level. Conducting exercise until exhaustion led to an increase in oxidative stress, as indicated by elevated levels of thiobarbituric reactive substances (TBARS) and reduced levels of glutathione, which make the body more vulnerable to oxidative stress, while high levels of GSH can help neutralizing ROS.^10^ Intense exercise with 80% of VO_2_max showed a decrease in the reduced form, GSH, which is important for maintaining the antioxidant defence system compared with moderate exercise (60% of VO_2_max), indicating that the effect of exercise on oxidative stress can be dose‐dependent.^11^ One theory is that intense exercise may lead to an initial increase in oxidative stress, which can stimulate the production of antioxidants as a protective mechanism. This process, known as hormesis, may ultimately lead to a decrease in oxidative stress over time. However, more research is needed to fully understand the relationship between intense exercise and oxidative stress and to determine how it may vary in different intensities. In this context, some studies revealed a significant increase in markers of oxidative stress and lipid oxidation such as malondialdehyde (MDA) immediately after exercise in low, moderate, and high intensity.^12^ Particularly, studies have shown that high‐intensity exercise can lead to increased oxidative stress in individuals who are both physically active and inactive.^10^, ^13^ During exercise, cells produce increased amounts of ROS as a result of increased oxygen demand and metabolism. Glutathione (GSH) is a particularly important antioxidant which plays a key role in neutralizing ROS. However, intense exercise can lead to a temporary increase in ROS production that exceeds the capacity of the antioxidant defence systems for neutralization, which in turn leads to a temporary decrease in GSH and other antioxidants as they are consumed in the process of neutralizing ROS.^10^, ^14^ GSH and antioxidant capacity will generally return to homeostasis following a period of rest and repair after intense exercise completion as reported in some studies, which is around 30 min.^14^, ^70^ In summary, moderate‐intensity exercise has been shown to be associated with a decrease in oxidative stress and an improvement in antioxidant status. However, very high intensity or prolonged endurance exercise may lead to an increase in oxidative stress in the short term due reactive oxygen species (ROS) production. The effect of exercise intensity on oxidative stress may also depend on the baseline antioxidant status in different subjects and other factors such as diet and lifestyle. For example, individuals who have a higher baseline antioxidant status may be less susceptible to oxidative stress during exercise, while those with a lower baseline antioxidant status may be more susceptible. Therefore, more research is needed to fully understand the relationship between exercise intensity and oxidative stress and to determine the optimal exercise dose for reducing oxidative stress in different individuals.

### 4.2 The impact of exercise on pro‐inflammatory cytokine production is complex and varies depending on intensity and duration

The immunomodulatory effect of exercise is well established, in which physical and psychosocial demands during intense exercise can initiate a stress response resulting in the release of inflammatory cytokines as well as catabolic and stress hormones.^71^ Exercise has been shown to affect the immune system and modulate the production of various cytokines, including both pro‐inflammatory and anti‐inflammatory cytokines. The immediate increase in pro‐inflammatory cytokines after exercise is thought to be a normal physiological response to the stress of exercise and is usually a transient level, activating the sympathetic‐adrenomedullary and hypothalamus–pituitary–adrenal (HPA) axes, which eventually returns to baseline levels within a few hours of exercise. Moreover, different intensity and duration of exercise can exhibit differences in the inflammatory response.^72^ Interestingly, the effect of exercise on pro‐inflammatory cytokine production are complex and depends on various factors, including the intensity and duration of the exercise, the fitness level, and the presence of preexisting health issues. In this context, intense or prolonged exercise may lead to an excessive production of pro‐inflammatory cytokines, known as ‘exercise‐induced inflammation’. Additionally, acute bouts of muscle strengthening exercises such as isokinetic, eccentric and knee extensor were reported to increase plasma and muscle interleukin (IL)‐6, IL‐8 and tumour necrosis factor alpha (TNF‐α) levels. Similarly, prolonged exercise, especially endurance exercise, has been shown to lead to a persistent increase in pro‐inflammatory cytokines, which is pronounced in untrained individuals or those who are older or have underlying health conditions. This suggests that long‐term exercise can modulate inflammatory and oxidative stress markers. The long‐term effects of this increase in cytokines on inflammation and immune function are not fully understood, and more research is needed to determine the optimal duration and intensity of exercise for reducing inflammation. In another study Stewart et al found that a 12‐week aerobic exercise training did not alter plasma IL‐6 and IL‐1β when comparing young active groups to older ones,^17^ suggesting that short‐term training may not be sufficient to induce changes in pro‐inflammatory cytokines. In a similar manner, Barry et al. demonstrated that short‐term (2 weeks) high‐intensity interval training and moderate‐intensity training did not change levels of circulating IL‐10, IL‐6 or TNF‐α in obese individual.^73^ In the same way, strength training for 6–12 weeks did not alter plasma IL‐1β, IL‐2, IL‐6 and TNF‐α concentration in healthy elderly adults and patients with chronic‐degenerative diseases, while 12 weeks of resistance training decreased muscle TNF‐α mRNA in frail elderly individuals.^74^ Short‐term endurance training at moderate intensities and the combination of endurance, strength, balance and flexibility training has been shown to increase plasma IL‐10 and reduce plasma IL‐6 and TNF‐α in healthy elderly adults.^74^ Presumably, the combination of different types of training may provide a comprehensive stimulus to the immune system, leading to increased IL‐10 production. Therefore, acute bouts of endurance exercise and short‐term chronic exercise training could be considered as appropriate methods to enhance mucosal immune function, reduce systemic markers of inflammation and promote anti‐inflammatory processes in elderly individuals.^74^ Findings from other studies have also suggested that cytokine levels vary significantly among athletes who belong to different sport disciplines, including higher anti‐inflammatory interleukin IL‐10 in moderate power and endurance compared with the high power and endurance counterparts, and higher SOD and MDA levels in the high‐power group than lower power counterpart.^18^ Overall, the effects of exercise on cytokine production are complex and multifaceted, and more research is needed to fully understand the mechanisms underlying these effects.

### 4.3 Exercise counteracts the detrimental effects of ageing and inflamm‐ageing

Recently, there has been a growing interest in the relationship between immune function and ageing as immune cells tend to decrease in both number and function with age, often resulting in a chronic inflammatory state.^75^ Exercise has been shown to have a number of beneficial effects on cellular ageing. Specifically, it has been suggested that exercise may have anti‐ageing effects on the immune system, the precise mechanisms and extent of these effects are not yet fully understood. Further research is needed to fully elucidate the relationship between exercise and immune ageing including the suggested exercise‐mediated anti‐immunosenescence, where exercise may have protective effects on the immune system by reducing the negative effects of ageing including telomeres shortening, mitochondrial function decline and chronic inflammation.^76^, ^77^ Specifically, advanced age is accompanied by a chronic low‐level systemic inflammatory known as ‘inflamm‐ageing’,^78^ which is characterized by increased production of pro‐inflammatory cytokines such as IL‐1, IL‐6, TNF‐α as well as C‐reactive protein (CRP).^78^ Elevated levels of pro‐inflammatory cytokines in the elderly are linked to a higher risk of morbidity and mortality, muscle wasting (sarcopenia) and frailty,^79^ while the precise cellular and molecular mechanisms leading to these alterations have not yet been identified. Several proposed explanations for these changes have been highlighted including oxidative stress, persistent DNA damage, ageing of stem cells and inhibition of autophagy through the activation of the inflammasome.^80^ Preliminary data suggest that telomere length increases with sport intensity, particularly in young athletes and is linked to higher levels of pro‐inflammatory cytokines, possibly indicating reduced ageing in high‐intensity sports with heightened immune response.^81^ Previous studies have shown that age‐dependent pro‐inflammatory or oxidative cellular environment could promote telomere attrition, cellular senescence and diseases that develop or become more prevalent with ageing.^82^ However, healthy diet and exercise training were shown to reduce the rate of telomere shortening during the ageing process.^83^ Studies have also demonstrated that regular engagement in exercise is associated with longer telomeres and may attenuate telomere attrition.^84^ An important consideration when interpreting the results of these studies is that they were limited by investigating the effects of training on telomere length (TL) based on questionnaire assessment of physical activity.^85^ Despite this, it is not yet clear what the most effective exercise recommendations are for maintaining telomere length. Accordingly, future research should aim to investigate the relationship between telomere length, exercise training and circulating inflammatory cytokine levels in order to understand the link between exercise, immune response and ageing.^85^ Further research is needed to fully elucidate the relationship between exercise and immune ageing including the suggested exercise‐mediated anti‐immunosenescence, where exercise may have protective effects on the immune system by reducing the negative effects of ageing including telomeres shortening, mitochondrial function decline and chronic inflammation.

### 4.4 Exercise‐induced changes in metabolic pathways: implications for insulin sensitivity

**FIGURE 2:**
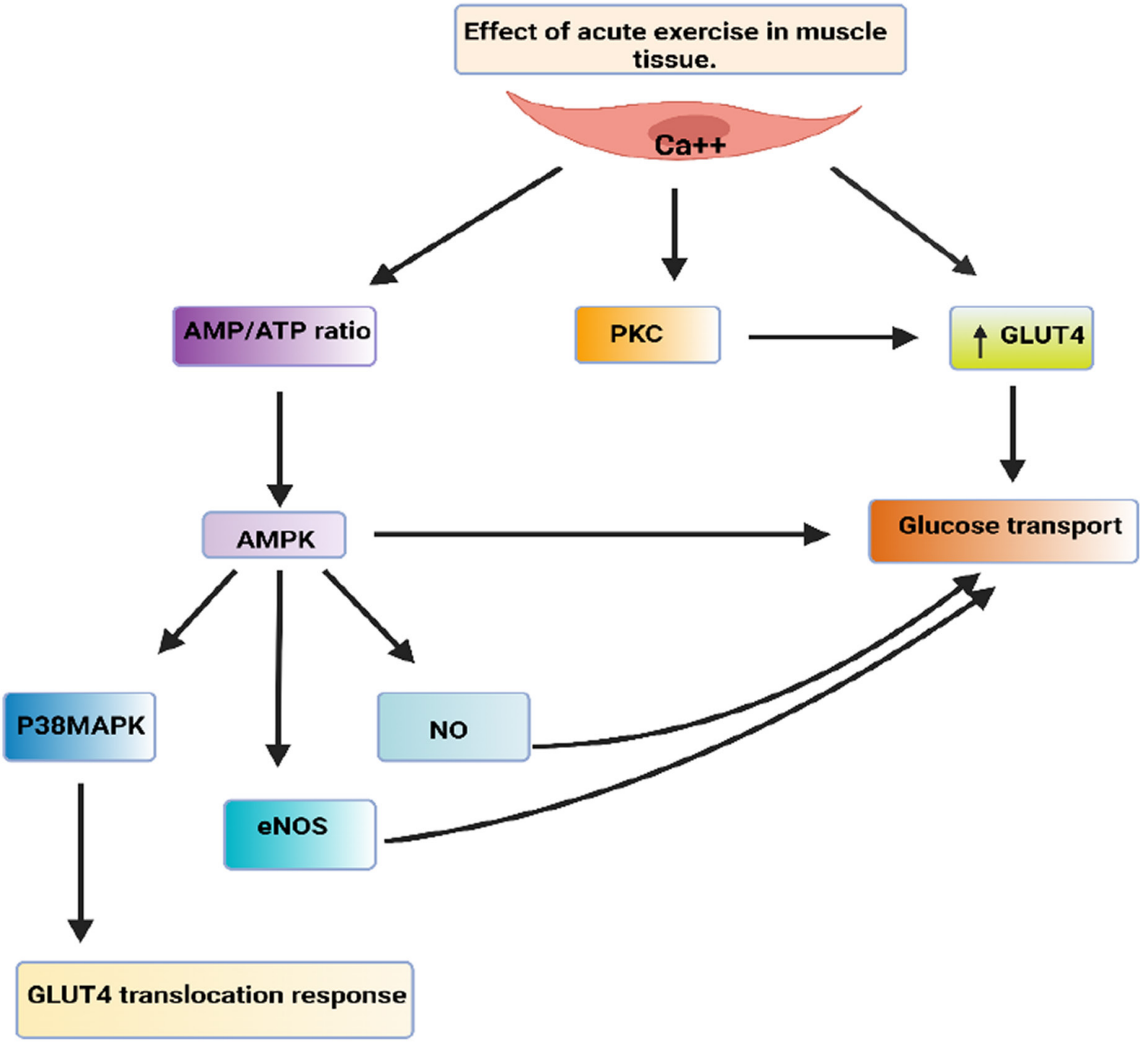
Mechanism by which acute exercise improves insulin sensitivity in muscle tissue and the involved pathways including nitric oxide (NO), nitric oxide synthase (NOS), activated protein kinase (MAPK) p38, 5′AMP‐activated protein kinase (AMPK), protein kinase C PKC.

Physical activity has been shown to have a beneficial effect on insulin sensitivity through multiple adaptations in glucose transport and metabolism. Glucose uptake can be increased for up to 2 hours after exercise, due to insulin‐independent mechanisms. These mechanisms include an increase in the amount of the glucose transporter protein GLUT4 associated with the plasma membrane and T‐tubules in muscle cells, which is induced by contraction, ultimately improving glucose metabolism. In diabetic patients, regular exercise has been shown to increase the expression of a protein called GLUT4 by 22%. This increase is accompanied by increased expression of Akt protein which upon phosphorylation inhibits the activity of GSK‐3β. This inhibition leads to the activation of gene transcription and protein synthesis, which may contribute to the improved insulin sensitivity observed following chronic exercise.^86^ Figure 2 illustrates the intricate mechanism through which acute exercise enhances insulin sensitivity in muscle tissue. It highlights the key pathways involved, encompassing nitric oxide (NO), nitric oxide synthase (NOS), activated protein kinase (MAPK) p38, 5′AMP‐activated protein kinase (AMPK) and protein kinase C (PKC). It is therefore assumed that the improvement in insulin sensitivity in response to exercise may be largely due to the regulation of transcriptional processes. Other adaptations include an increase in GLUT4 translocation to the plasma membrane, increased insulin‐stimulated glucose uptake, increased glycogen synthesis and an increase in hepatic glucose uptake. Studies have demonstrated that exercise can also lead to an increase in the activity of enzymes involved in glucose metabolism, such as hexokinase, glucokinase and phosphofructokinase, as well as an increase in the activity of enzymes involved in fatty acid oxidation. Furthermore, exercise can lead to an increase in adiponectin levels, which can stimulate glucose uptake and fatty acid oxidation. These combined effects of exercise on glucose and lipid metabolism can ultimately lead to improved insulin sensitivity. Evidence indicates that exercise can also lead to changes in how the body processes lipids and can improve the regulation of glucose production in the liver. These changes may be due to increased activation of insulin receptor substrate 1(IRS‐1), which activates Phosphatidylinositol 3‐Kinase (PI3‐K) signalling pathway, leading to improved insulin sensitivity and signalling without necessarily altering protein levels.^87^

Previous studies have shown that exercise can produce varying metabolic changes depending on its intensity and duration.^88^, ^89^ According to recent findings, athletes with high power (resistance) and endurance (aerobic) capabilities have unique metabolic profiles related to steroid hormone production and fatty acid metabolism.^81^ These metabolic changes include alterations in sex steroid hormone concentrations such as testosterone metabolites androstenediol 3‐alpha, 17‐alpha and etiocholanolone glucuronide (ADT‐G), as well as long‐chain fatty acids, diacylglycerols and fatty acid carnitines.^81^ ADT‐G, a metabolite formed during the metabolism of testosterone or dihydrotestosterone (DHT), is a major contributor to total androgen glucuronides in women, accounting for approximately 93%.^90^ Elevated levels of ADT‐G in relation to dehydroepiandrosterone sulphate (DHEAS) have been identified as a potential marker of insulin resistance in obese women with polycystic ovary syndrome (PCOS),^91^ and ADT‐G has also been suggested as a potential biomarker for peripheral hyperandrogenism, or excess of androgens, in adult women with acne.^92^ A recent study has found that certain metabolites, including androgenic steroids, long‐chain fatty acids and microbiota‐produced compounds, are associated with insulin resistance in young, non‐obese females in Qatar, when compared to those without insulin resistance.^93^ Both physically active healthy athletes and sedentary non‐obese individuals with insulin resistance (IR) share some metabolic pathways that are related to exercise and insulin sensitivity. These pathways involve steroidogenesis and glucuronidation, which is the process of adding a molecule called glucuronic acid.

### 4.5 Exercise alters steroid hormone production, leading to positive health outcomes and improved muscle strength and insulin sensitivity

Exercise has been demonstrated to alter steroid hormone production in various ways. For example, physical activity can increase the production of testosterone and DHEA (dehydroepiandrosterone) hormones, while it can also decrease the production of other steroids, such as cortisol, which has been linked to a number of positive health outcomes, including weight loss and the enhancement of the immune cells function. Moreover, according to recent reports, exercise can increase the ratio of testosterone to cortisol, which may be beneficial for muscle strength and insulin sensitivity.^94^, ^95^ Steroids are often eliminated from the body through a process called glucuronidation, which involves the transfer of a polar moiety from UDP‐glucuronate to steroids such as androsterone in various steroid target tissues and the liver. ADT‐G is formed from androsterone by UDP‐glucuronosyltransferases, with the major enzymes being UDP‐glucuronosyltransferases (UGTs) 2B15 and 2B17.^96^ The UGT superfamily is divided into two sub‐families, UGT1 and UGT2, based on sequence homology. Currently, there are four known UGT2B proteins (2B4, 2B7, 2B15 and 2B17), that can target glucuronidation of steroid molecules.^97^ Genetic variants in UGT enzymes were reported to be associated with alterations in total testosterone levels and insulin sensitivity.^98^ A deletion polymorphism in UGT2B17 gene was previously associated with a decrease in the activity of the enzyme UDP‐glucuronosyltransferase, which is responsible for the formation of glucuronides. As a result, individuals with this polymorphism may experience lower levels of glucuronidation, leading to increased insulin sensitivity.^98^

## 5 SUMMARY

Physical activity has been shown to have a beneficial effect on insulin sensitivity, both in normal and IR subjects. Several recent studies have demonstrated that increasing physical activity and cardiorespiratory fitness can improve high waist circumference, dyslipidaemia, hypertension and insulin resistance. Furthermore, recent evidence suggests that physical activity may improve insulin sensitivity and reduce the risk of insulin resistance‐related complications, such as T2D. There are several molecular pathways that are activated or inhibited by exercise in non‐obese IR individuals, some of these pathways have been identified as being shared between exercise and insulin sensitivity including glucose uptake and utilization, lipid metabolism, mitochondrial function, inflammation, autophagy, oxidative stress, neuroendocrine signalling and microbiome. Overall, the intensity, duration and type of exercise can all influence the molecular changes that occur in non‐obese individuals and the relationship between these changes and insulin sensitivity. Furthermore, higher‐intensity exercise has been shown to have a greater impact on molecular pathways than lower‐intensity exercise. For example, high‐intensity interval training (HIIT) and longer‐duration exercise has been shown to increase insulin sensitivity more than moderate‐intensity continuous training (MICT) and shorter‐duration exercise, respectively. However, the optimal duration of exercise for improving insulin sensitivity may vary depending on the intensity and type of exercise. Also, different types of exercise can affect molecular pathways in different ways. For example, resistance training has been shown to increase insulin sensitivity more than aerobic exercise in non‐obese individuals.^99^, ^100^ Future research should focus on understanding the genetic and epigenetic factors that may influence the molecular and cellular responses to exercise in non‐obese individuals, and how do these factors impact insulin sensitivity. Another direction for future research could be to compare the molecular changes induced by exercise in non‐obese individuals to those observed in obese individuals. Additional research is also needed to assess whether these molecular regulators induced by exercise can be replicated or enhanced through the use of pharmacological or dietary interventions in non‐obese individuals, and how do these interventions affect insulin sensitivity.

## AUTHOR CONTRIBUTIONS

**Shamma Almuraikhy:** Conceptualization (equal); writing – original draft (lead). **Asmaa Doudin:** Writing – review and editing (equal). **Alexander Domling:** Supervision (equal); writing – review and editing (equal). **Asmaa Althani:** Supervision (equal); writing – review and editing (equal). **Mohamed Elrayess:** Conceptualization (equal); supervision (equal); writing – review and editing (equal).

## FUNDING INFORMATION

This research was funded by Qatar University grant number IRCC‐2022‐467 (M.A.E).

## CONFLICT OF INTEREST STATEMENT

The authors declare no conflict of interest.

## Data Availability

Not applicable.
